# Human umbilical cord mesenchymal stem cells-derived extracellular vesicles facilitate the repair of spinal cord injury via the miR-29b-3p/PTEN/Akt/mTOR axis

**DOI:** 10.1038/s41420-021-00572-3

**Published:** 2021-08-11

**Authors:** Xiao Xiao, Weiwei Li, Dingchao Rong, Zhenchao Xu, Zhen Zhang, Hongru Ye, Liqiong Xie, Yunqi Wu, Yilu Zhang, Xiyang Wang

**Affiliations:** 1 Department of Spine Surgery, The Xiangya Hospital of Central-South University, Changsha, China; 2 Hunan Engineering Laboratory of Advanced Artificial Osteo-materials, Changsha, China

**Keywords:** Cellular neuroscience, Stem-cell research

## Abstract

Spinal cord injury (SCI) is a salient traumatic disease that often leads to permanent disability, and motor and sensory impairments. Human umbilical cord mesenchymal stem cells (HucMSCs) have a wide application prospect in the treatment of SCI. This study explored the repair effect of HucMSCs-derived extracellular vesicles (HucMSCs-EVs) on SCI. HucMSCs and HucMSCs-EVs were cultured and identified. The rat model of SCI was established, and SCI rats were treated with HucMSCs-EVs. The motor function of SCI rats and morphology of spinal cord tissues were evaluated. Levels of NeuN, GFAP, and NF200 in spinal cord tissues were detected and cell apoptosis was measured. SCI rats were treated with EVs extracted from miR-29b-3p inhibitor-transfected HucMSCs. The downstream gene and pathway of miR-29b-3p were examined. HucMSCs-EVs-treated rats showed obvious motor function recovery and reduced necrosis, nuclear pyknosis, and cavity. HucMSCs-EVs alleviated spinal cord neuronal injury. miR-29b-3p was poorly expressed in SCI tissues, but highly expressed in EVs and SCI rats treated with EVs. miR-29b-3p targeted PTEN. Inhibition of miR-29b-3p or overexpression of PTEN reversed the repair effect of EVs on SCI. EVs activated the AKT/mTOR pathway via the miR-29b-3p/PTEN. In conclusion, HucMSCs-EVs reduced pathological changes, improved motor function, and promoted nerve function repair in SCI rats via the miR-29b-3p/PTEN/Akt/mTOR axis.

## Introduction

Spinal cord injury (SCI) is a perplexing traumatic disease that often leads to permanent disability, as well as motor and sensory impairments [1]. Statistics show that about 2.5 million patients are suffering from SCI in the world, with approximately 130,000 newly diagnosed cases reported each year [2]. SCI not only ruins the life quality of patients physically and psychologically but also brings heavy social and economic burdens to patients [3]. The major pathologic hallmarks associated with secondary damage of SCI include inflammation, oxidative stress, necrosis, and neuronal apoptosis [4]. To be specific, about 45% of SCI patients suffer severe neurological loss, and complete or incomplete tetraplegia or even respiratory compromise can be seen in some cases [5]. At present, the treatment approaches for SCI patients mainly include pharmacological agents, surgical treatment, and cell therapy [6, 7].

Human umbilical cord mesenchymal stem cells (HucMSCs) possess the advantages of multilineage differentiation, short proliferation time, easy extraction, and long survival time after transplantation, which are accepted as favorable seed cells for transplantation [8]. As a promising source of mesenchymal stem cells (MSCs), HucMSCs are demonstrated to play a critical part in the management of SCI [9]. For example, transplantation of HucMSCs can decrease the expression of IL-7 and facilitate the polarization of M2 macrophages, thus accelerating the repair of the injured site and ameliorating the motor function of SCI mice [10]. Extracellular vesicles (EVs) are cell-derived microparticles that exist in body fluids, including microbubbles, exosomes, and apoptotic bodies [11]. It has been reported that EVs derived from MSCs exhibit the same therapeutic effect as parental cells [12]. Nevertheless, the repair mechanism of HucMSCs-EVs in SCI has not been completely understood.

Mechanically, EVs occupy a crucial part in intercellular communication via transferring microRNAs (miRs) [13]. The ability of miRs to modulate cell state and function via post-transcriptionally silencing genes is being considered as an important factor in the pathophysiology of SCI [14]. MiR-29b-3p has recently been reported to play a regulatory role in malignancies [15], osteoarthritis [16], and cardiac fibrosis formation [17]. Intriguingly, Liu et al. have revealed that injection of miR-29b mimic into the SCI site can rescue neuron death and eliminate the apoptosis induced by SCI [18]. At present, whether HucMSCs-EVs can promote the repair of SCI by carrying miR-29b-3p remains unclear. This study herein investigated the effect of HucMSCs-EVs on the repair of SCI, along with its underlying mechanism, which shall shed light on the cell therapy for SCI.

## Results

### Identification of HucMSCs and EVs

**Fig. 1:**
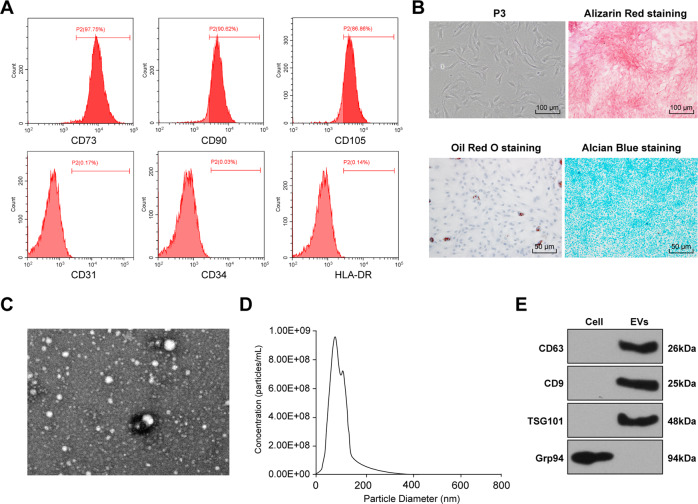
Identification of HucMSCs and HucMSCs-EVs. **A** Surface markers of HucMSCs: CD73, CD90, and CD105 (positive markers), and CD31, HLA-DR, and CD34 (negative markers) were detected using flow cytometry. **B** Morphology of HucMSCs was observed under a light microscope (left); osteogenesis, adipogenesis, and chondrogenesis were observed using alizarin red staining, oil red O staining, and alcian blue staining respectively. **C** the ultrastructure of HucMSCs-EVs was observed by TEM. **D** the particle size of EVs was detected using Nanosight analysis. **E** the surface marker proteins of EVs (CD63, CD9, and TSG101) were detected using Western blotting. The experiment was repeated three times.

MSCs-derived EVs facilitate functional recovery of SCI rats by reducing inflammation [19]. Nevertheless, the effect of HucMSCs-EVs on the recovery of SCI remains unknown. The surface antigens of HucMSCs at passage 3 were identified using flow cytometry. HucMSCs were positive for CD73, CD90, and CD105, while negative for CD31, CD34, and HLA-DR (Fig. 1A). Additionally, HucMSCs showed favorable osteogenic, adipogenic, and chondrogenic differentiation abilities (Fig. 1B). These results demonstrated that HucMSCs were isolated successfully. Then, EVs were isolated from HucMSCs. The EVs were round or oval with complete envelope structure and low-density substances, showing the typical morphology of EVs (Fig. 1C). The results of NTA showed that the particles presented multimodal distribution, and most of the particles were in the range of EVs diameter (30–150 nm) (Fig. 1D). Furthermore, Western blotting showed that CD63, CD9, and TSG101 were evidently expressed on EVs, but not GRP94 (Fig. 1E). These results demonstrated that EVs were isolated successfully.

### HucMSCs-EVs promoted the repair of SCI in rats

**Fig. 2:**
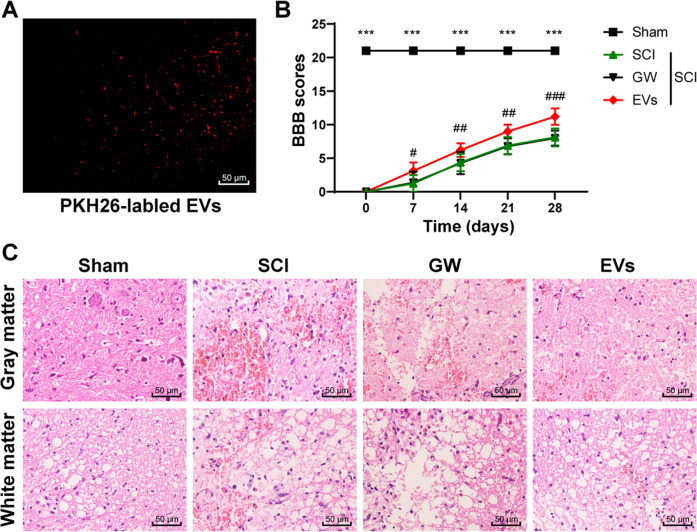
HucMSCs-EVs repair SCI in rats. The rat model of SCI was established, and rats were treated with EVs. The conditioned medium of HucMSCs treated with GW4869 was used as a control. **A** PKH26-labeled EVs in spinal cord tissues of rats were detected using immunofluorescence. **B** the motor function of rats in each group was evaluated by BBB scale on the 0, 7th, 14th, 21st, 28th day after the operation. **C** the pathological changes of spinal cord tissues were measured using HE staining on the 28th day after the operation. *N* = 6. Data are expressed as mean ± standard deviation and analyzed using two-way ANOVA, followed by Tukey’s multiple comparisons test, ***p < 0.001, Sham vs. SCI; ^###^*p* < 0.001, GW vs. EVs.

The rats were treated with PHK26-labeled EVs, and the labeled EVs could be observed under the microscope (Fig. 2A). Then the rat model of SCI was established. The results of the BBB scale showed that compared with the sham-operated rats, the SCI rats showed a functional loss in the hind limbs and mainly relied on forelimb movement with a significantly decreased BBB score (Fig. 2B; *p* < 0.05). After SCI rats were injected with HucMSCs-EVs, the behavior recovery was evaluated by BBB scale. The behavior of rats in the EVs group changed significantly on the 7th day after SCI compared with that in the GW group (*p* < 0.05). From the 14th day to the 28th day, SCI rats in the EVs group also showed the advantage of motor function recovery (Fig. 2B; all *p* < 0.001). On the 28th day, the white matter and gray matter of the spinal cords were gradually destroyed, and cavities appeared in SCI rats, while EVs-treated rats showed reduced necrosis, nuclear pyknosis, and cavity (Fig. 2C). These results suggested that HucMSCs-EVs could repair SCI in rats.

### HucMSCs-EVs alleviated spinal cord neuronal injury in rats

**Fig. 3:**
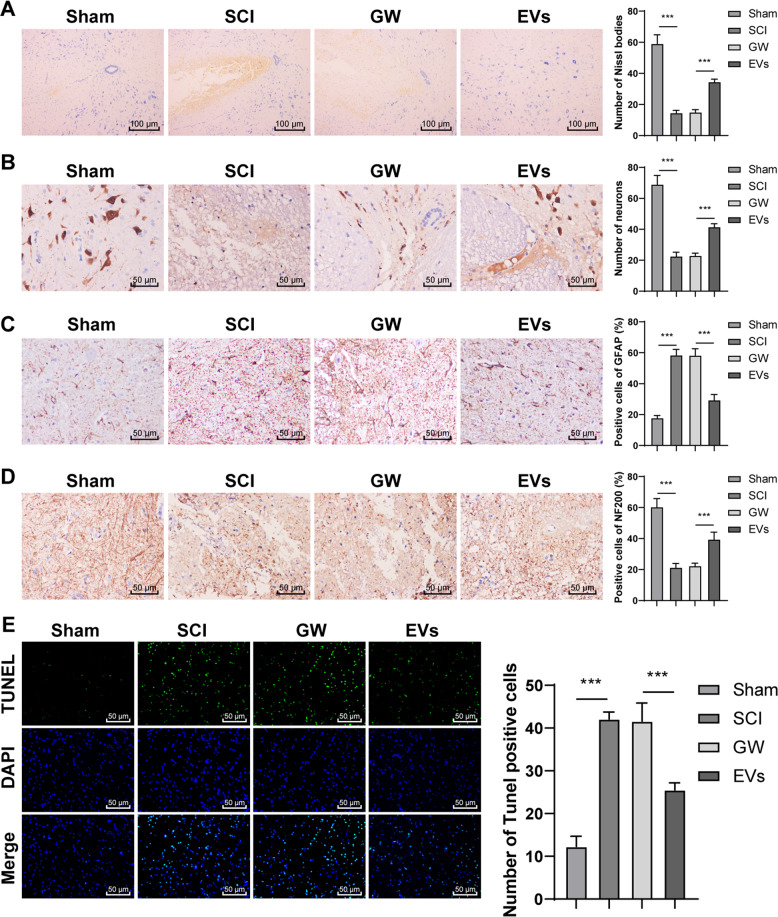
HucMSCs-EVs alleviate spinal cord neuronal injury in rats. **A** The rat model of SCI was established, and rats were treated with EVs. The conditioned medium of HucMSCs treated with GW4869 was used as a control. **A** Nissl bodies in SCI rats were detected using Nissl staining on the 28th day after the operation. **B**–**D** Expressions of NeuN, GFAP, and NF200 were detected using immunohistochemistry. **E** Apoptosis in spinal cord tissues of SCI rats was detected using TUNEL on the 28th day after the operation. *N* = 6. Data are expressed as mean ± standard deviation and analyzed using one-way ANOVA, followed by Tukey’s multiple comparisons tests, ***p < 0.001.

SCI is also a nerve injury caused by external mechanical injury [20]. We further explored the protective effect of EVs against spinal cord neuronal injury on rats. There were fewer Nissl bodies in the SCI rats than in the sham-operated rats, while the EVs-treated rats showed an increase in Nissl bodies and regeneration of injured axons (Fig. 3A; all *p* < 0.001). NeuN, GFAP, and NF-200 represent neurons, axon regeneration, and glial scab formation in the spinal cord [21, 22]. The number of NeuN was decreased significantly in SCI rats but increased in the EVs-treated rats (Fig. 3B; all *p* < 0.001). The EVs-treated rats showed decreased GFAP expression, regularly arranged nerve fibers, and increased NF200 expression (Fig. 3C/D; all *p* < 0.001). In addition, apoptosis plays an important role in the progressive degeneration of SCI [23]. TUNEL staining was applied to measure cell apoptosis, and the results showed that compared with those in the sham-operated rats, TUNEL-positive cells in the SCI rats were increased significantly 28 days after SCI. EVs treatment notably decreased the number of TUNEL-positive cells (Fig. 3E; *p* < 0.001). These results suggested that EVs could alleviate spinal cord neuronal injury in rats.

### HucMSCs-EVs promoted the repair of SCI by carrying miR-29b-3p

**Fig. 4:**
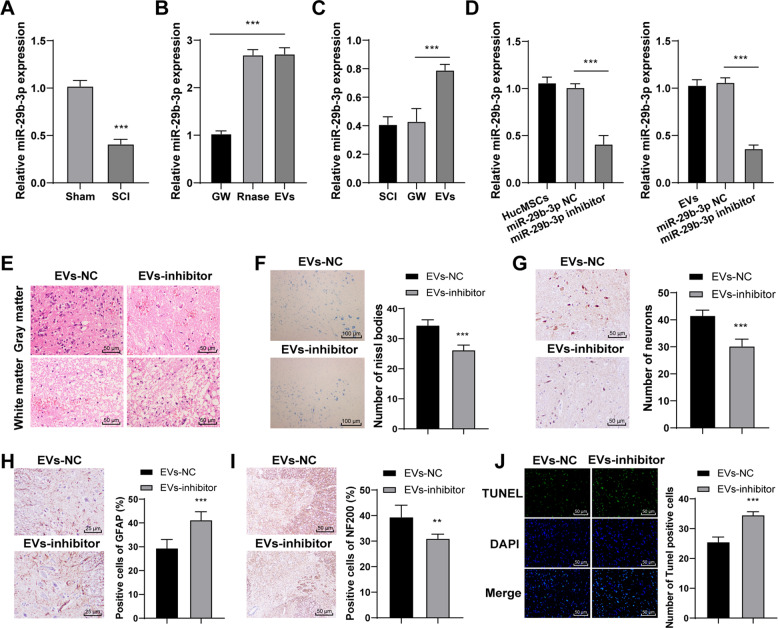
HucMSCs-EVs repair SCI in rats by carrying miR-29b-3p. **A** miR-29b-3p expression in spinal cord tissues of SCI rats was detected using RT-qPCR on the 28th day after operation. **B** miR-29b-3p expression in EVs was detected using RT-qPCR. **C** miR-29b-3p expression in spinal cord tissues of EVs-treated SCI rats was detected using RT-qPCR on the 28th day after the operation. **D** miR-29b-3p expression in miR-29b-3p inhibitor-treated HucMSCs and EVs was detected using RT-qPCR. **E** pathological changes were measured using HE staining. **F** Nissl bodies were detected using Nissl staining. **G**–**I** Expressions of NeuN, GFAP, and NF200 were detected using immunohistochemistry. **J** Apoptosis in spinal cord tissues of SCI rats was detected using TUNEL. *N* = 6. Data are expressed as mean ± standard deviation and analyzed using *t*-test or one-way ANOVA, followed by Tukey’s multiple comparisons test, **p < 0.01, ***p < 0.001.

EVs can play a role as critical agents of cell-to-cell communication by carrying miRs [24, 25]. Previous literature has revealed that exosomes secreted from miRNA-29b-modified mesenchymal stem cells can alleviate traumatic spinal cord injury in rats [26]. We speculated that miR-29b-3p carried by HucMSCs-EVs might play a role in SCI. Hence, we detected miR-29b-3p expression in spinal cord tissues of rats on the 28th day after SCI operation. RT-qPCR showed that miR-29b-3p was poorly expressed in spinal cord tissues of SCI rats (Fig. 4A; all *p* < 0.001). Then, miR-29b-3p expression in EVs and in EVs-treated SCI rats was detected. The results exhibited that miR-29b-3p expression was upregulated in EVs (Fig. 4B; all *p* < 0.001) and in spinal cord tissues of EVs-treated SCI rats (Fig. 4C; all *p* < 0.001). These results suggested that EVs carried miR-29b-3p into the spinal cord tissues of rats. After that, we explored the effect of miR-29b-3p on SCI repair. HucMSCs were transfected with miR-29b-3p inhibitor, and the transfection efficiency was confirmed using RT-qPCR. Then, EVs were extracted from miR-29b-3p inhibitor-transfected HucMSCs. miR-29b-3p expression was notably decreased in miR-29b-3p inhibitor-transfected EVs (Fig. 4D). HE staining revealed that the protective effect of EVs-inhibitor on the spinal cord was significantly reduced (Fig. 4E). Moreover, Nissl bodies and NeuN of rats in the EVs-inhibitor group were reduced in comparison with those in the EVs-NC group (Fig. 4F/G; all *p* < 0.001). The rats in the EVs-inhibitor group showed increased GFAP expression and decreased NF200 expression (Fig. 4H/I; all *p* < 0.001). The cell apoptosis of SCI rats in the EVs-inhibitor group was also notably promoted (Fig. 4J; all *p* < 0.001). These results indicated that HucMSCs-EVs carried miR-29b-3p to promote the repair of SCI in rats.

### miR-29b-3p targeted PTEN in spinal cord tissues of rats

**Fig. 5:**
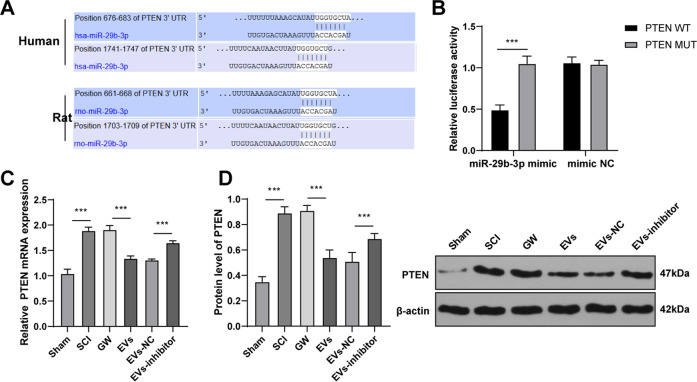
miR-29b-3p targets PTEN. **A** The binding site between miR-29b-3p and PTEN in humans and rats was predicted by Targetscan. **B** The binding relationship between miR-29b-3p and PTEN was verified using a dual-luciferase reporter gene assay. **C**/**D** PTEN expression in spinal cord tissues of rats in each group was detected using RT-qPCR and Western blotting. *N* = 6. Data are expressed as mean ± standard deviation and analyzed using one-way ANOVA or two-way ANOVA, followed by Tukey’s multiple comparisons tests, ***p < 0.001.

It has been reported that PTEN is a negative regulator of SCI [27] and could block neurogenesis and axonal regeneration [28]. Targetscan database (http://www.targetscan.org/vert_71/) showed that there is the same specific binding region between PTEN 3′ UTR and miR-29b-3p sequence in humans and rats (Fig. 5A). The binding relationship between PTEN and miR-29b-3p was verified using a dual-luciferase reporter gene assay (Fig. 5B; all *p* < 0.001). Therefore, we speculated that miR-29b-3p carried by HucMSCs-EVs participated in SCI by targeting PTEN in rats. PTEN expression in spinal cord tissues of SCI rats on the 28th day after SCI was detected. RT-qPCR and Western blotting showed that PTEN was highly expressed in SCI rats (Fig. 5C/D; all *p* < 0.001). Moreover, PTEN expression in EVs- and EVs-inhibitor-treated SCI rats was detected. The results exhibited that EVs treatment significantly reduced PETN expression, while EVs-inhibitor treatment promoted PETN expression in spinal cord tissues of SCI rats (Fig. 5C/D; all *p* < 0.001). These results suggested that miR-29b-3p carried by HucMSCs-EVs targeted PTEN in rats.

### Overexpressing PTEN reversed the repair effect of HucMSCs-EVs on SCI rats

**Fig. 6:**
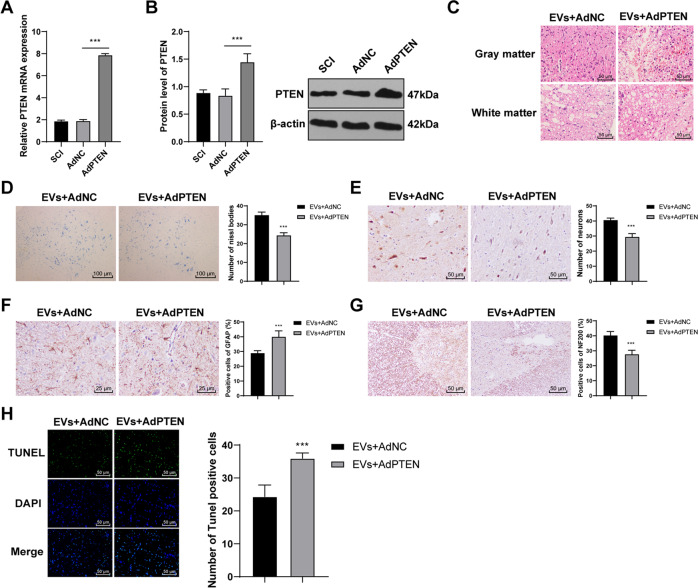
Overexpressing PTEN reverses the repair effect of HucMSCs-EVs on SCI rats. **A**, **B** The transfection efficiency of PTEN adenovirus was detected using RT-qPCR and Western blotting. SCI rats were co-treated with HucMSCs-EVs and PTEN adenovirus. **C** Pathological changes were measured using HE staining. **D** Nissl bodies were detected using Nissl staining. **E**–**G** expressions of NeuN, GFAP, and NF200 were detected using immunohistochemistry. **H** Apoptosis in spinal cord tissues of SCI rats was detected using TUNEL. *N* = 6. Data are expressed as mean ± standard deviation and analyzed using and analyzed using *t* test or one-way ANOVA, followed by Tukey’s multiple comparisons tests, ***p < 0.001.

To verify the role of PTEN in the repair of SCI in rats, we infected SCI rats with AdPTEN. The infection efficiency was confirmed using RT-qPCR and Western blotting (Fig. 6A/B; all *p* < 0.001). SCI rats were co-treated with AdPTEN and EVs. HE staining showed that the protective effect of EVs + AdPTEN on the spinal cord was significantly reduced (Fig. 6C). Nissl bodies and NeuN of spinal cord tissues in the EVs + AdPTEN group were reduced (Fig. 6D/E; all *p* < 0.001). The rats in the EVs + AdPTEN group showed increased GFAP expression and decreased NF200 expression (Fig. 6F/G; all *p* < 0.001). The cell apoptosis of SCI rats in the EVs + AdPTEN group was also notably promoted (Fig. 6H; all *p* < 0.001). These results revealed that overexpressing PTEN reversed the repair effect of HucMSCs-EVs on SCI rats.

### HucMSCs-EVs activated the Akt/mTOR pathway during SCI repair in rats via the miR-29b-3p/PTEN axis

**Fig. 7:**
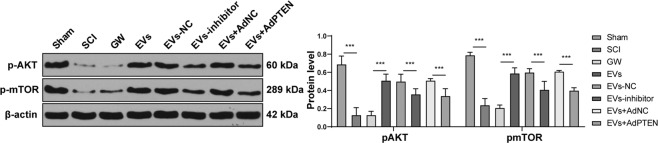
HucMSCs-EVs activate the Akt/mTOR pathway during SCI repair in rats via the miR-29b-3p/PTEN axis. The expression and phosphorylation of Akt and mTOR in the Akt/mTOR signaling pathway were detected using Western blotting. *N* = 6. Data are expressed as mean ± standard deviation and analyzed and analyzed using two-way ANOVA, followed by Tukey’s multiple comparisons tests, ***p < 0.001.

Previous literature has shown that PTEN inhibits the Akt/mTOR pathway in the functional recovery after SCI [29]. The levels of pAKT and pmTOR in SCI rats were reduced significantly and promoted after EV treatment. EVs-inhibitor and EVs + AdPTEN could increase the levels of pAKT and pmTOR in SCI rats (Fig. 7; all *p* < 0.001). These results indicated that HucMSCs-EVs activated the Akt/mTOR pathway during SCI repair in rats via the miR-29b-3p/PTEN axis.

## Discussion

SCI affects millions of population around the world and may result in paraplegia and quadriplegia [30]. The stem cell transplantation in the treatment of SCI has received widespread concern and has been demonstrated to bear a broad application prospect [31]. MSCs-EVs are reported to alleviate apoptosis and inflammatory response and facilitate angiogenesis after SCI [19]. Among MSCs, HucMSCs are not only readily available that can be collected in a non-invasive way but also have lower immunogenicity than stem cells from other sources [32, 33]. This study demonstrated that HucMSCs-EVs facilitated SCI repair in rats via the miR-29b-3p/PTEN/Akt/mTOR axis.

Notably, the immerse therapeutic potential of HucMSCs in the treatment of SCI has been unveiled [34]. HucMSCs-derived EVs were cultured and isolated in vitro and the rat model of SCI was established. It has been recognized that SCI can cause damages to neurons, resulting in extensive neurodegeneration and neuronal death [35]. The SCI rats were treated with HucMSCs-EVs and the EVs-treated rats showed obvious motor function recovery and reduced necrosis and nuclear pyknosis and cavity. Sun et al. have also indicated that HucMSCs-derived exosomes facilitate SCI healing via reducing the inflammatory response at the injury site [36]. Briefly, HucMSCs-EVs could repair SCI in rats.

As a marker of neuronal function, Nissl bodies are rich in neurons with a strong metabolic function. Nissl bodies may be decreased, disintegrated, and even disappeared when the neurons are damaged [37]. Spinal cord axon destruction can lead to a cascade of secondary harmful reactions gradually spreading to the adjacent tissues, thus resulting in the expansion of the lesion and deterioration of the disease [38]. There were fewer Nissl bodies in the SCI rats than in the sham-operated rats, while the EVs-treated rats showed an increase in Nissl bodies and regeneration of injured axons. NeuN is localized in the nuclei of mature neurons in the central nervous system of mammals and is acknowledged as a marker of mature neurons [39]. We showed that the number of NeuN was decreased significantly in SCI rats, but increased in the EVs-treated rats. GFAP and its decomposition products are released into biological fluids rapidly after SCI, which work as a candidate biomarker for neurological disorders [40]. NF-200, a neurofilament protein, is a biological marker of mature neurons in the central nervous system [41]. The EVs-treated SCI rats showed decreased GFAP expression, regularly arranged nerve fibers, and increased NF200 expression. In addition, the SCI-associated neuronal apoptosis process seriously affects the spinal cord function and causes secondary or permanent neuronal damages, which may lead to irreparable damage to the central nervous system [42]. Compared with those in the sham-operated rats, TUNEL-positive cells in the SCI rats were increased significantly 28 days after SCI. EVs treatment notably decreased the number of positive cells in the SCI rats. Stem cell therapy can induce the regeneration of damaged neuron tissues, promote the secretion of neuron factors, and enhance axon regeneration [43]. Following spinal cord compression injury, HucMSCs transplantation can facilitate functional recovery by enhancing anti-apoptotic and neuroprotective effects [44]. These results indicated that EVs could alleviate spinal cord neuronal injury in rats.

EVs can transfer macromolecular signals such as miRs to neighboring cells and alter their transcriptional activity [45]. Emerging evidence has revealed that deregulation of miRs is concerned with apoptosis, inflammatory response, functional recovery, and regeneration in SCI [46]. Elevated miR-29b expression has been demonstrated to repress neuronal apoptosis caused by spinal cord injuries [47] and ethanol neurotoxicity [48]. Consistently, we exhibited that miR-29b-3p was downregulated in spinal cord tissues of SCI rats, but upregulated in EVs and in spinal cord tissues of EVs-treated SCI rats. Moreover, HucMSCs were transfected with miR-29b-3p inhibitor, and the protective effect of HucMSCs-EVs inhibitor on the spinal cord was significantly reduced. Wan et al. have also exhibited that MSCs-EVs repress the fibroblast proliferation by carrying miR-29b-3p in idiopathic pulmonary fibrosis [49]. The co-treatment of human neuroepithelial stem cells and miR-29b mimic exerts therapeutic effects on SCI by suppressing PTEN expression [50]. All these results showed that HucMSCs-EVs carried miR-29b-3p to repair SCI in rats. Furthermore, MSCs-derived exosomal miR-29b promotes neuronal regeneration and alleviates histopathological damage, thus preventing traumatic spinal cord injury in rats [26]. The binding relationship between PTEN and miR-29b-3p was verified using dual-luciferase reporter gene assay in this study. PTEN is accepted as a negative regulator of SCI recovery [27], which can hinder neuritogenesis and axonal regeneration [28]. We found that PTEN was highly expressed in SCI rats and EVs treatment significantly reduced PETN expression. To verify the role of PTEN in the repair of SCI in rats, we infected SCI rats with AdPTEN and found that overexpressing PTEN reversed the repair effect of HucMSCs-EVs on SCI rats. PTEN silencing contributes to axonal regeneration of the corticospinal tract after SCI [51]. Knockdown of PTEN combined with overexpression of ChABC facilitates functional recovery in SCI rats [52]. Taken together, HucMSCs-EVs facilitated SCI repair by carrying miR-29b-3p, and overexpression of PTEN reduced the repair effect of HucMSCs-EVs on SCI rats.

Thereafter, we shift to investigating the downstream pathway modulated by miR-29b-3p/PTEN. Genetic knockout of PTEN can promote axon regeneration in the central nervous system after SCI, while inhibition of mTOR blocks the promoting effect of PTEN knockout [53]. mTOR is a downstream signaling molecule of Akt, which controls and coordinates nerve regeneration after SCI [54, 55]. We showed that the levels of pAKT and pmTOR in SCI rats were reduced significantly, but promoted after EVs treatment. EVs-inhibitor and EVs + AdPTEN could increase the levels of pAKT and pmTOR in SCI rats. Activating the mTOR can effectively relieve nerve tissue damages and secondary injury after SCI [56]. In brief, HucMSCs-EVs activated the Akt/mTOR pathway during the repair of SCI in rats via the miR-29b-3p/PTEN axis.

## Conclusion

To sum up, HucMSCs-EVs reduced pathological changes, improved motor function, and promoted nerve function repair in SCI rats via the miR-29b-3p/PTEN/Akt/mTOR axis. This fundamental information might offer a theoretical basis for the cell therapy of SCI in the clinical. In the future, we shall carry out more prospective trials on the feasibility and safety of HucMSCs-EVs in the treatment of SCI, so as to refine our clinical guidance.

## Materials and methods

### Ethics statement

All animal operations were in accordance with the operating standards of laboratory animals established by the animal ethics committee of the Xiangya Hospital of Central-South University. All efforts were made to minimize the animal suffering. Informed consent was signed by each umbilical cord donor.

### Isolation and identification of HucMSCs

The fresh umbilical cords were collected and processed within 6 h. The fresh umbilical cords were washed twice with phosphate-buffered saline (PBS) containing penicillin and streptomycin to remove the cord blood. Then the umbilical cords were cut into 1–2 mm pieces, floated in the low-glucose Dulbecco’s modified Eagle’s medium (DMEM) containing 10% fetal bovine serum (FBS), 5% human serum, and 1% penicillin and streptomycin (v/v), and cultured in humid air with 5% CO_2_ at 37 °C. Non-adherent cells were removed by PBS washing. The medium was refreshed every 3 days. After 10 days, the well-developed fibroblast-like cell colonies appeared. The tissue culture was trypsinized and passaged (undiluted) into a new culture dish for further amplification. The medium was refreshed every 3 days.

HucMSCs at passage 3 were detached with trypsin (Gibco by Life technologies, Grand Island, NY, USA) and resuspended in PBS (1 × 10^6^ cells/mL). Then, 200 μL cell suspension was added into EP tube and incubated at 37 °C in humidified air with 5% CO_2_. The expressions of CD73, CD90, CD105 (Abcam Inc., Cambridge, MA, USA), CD31, CD34, and HLA-DR (PE, eBioscience, San Diego, CA, USA) were analyzed using flow cytometry.

HucMSCs at passage 3 were detached with trypsin and prepared into a single-cell suspension. The cells were seeded to the 6-well plates (1 × 10^5^ cells/mL). The differentiation ability of HucMSCs into adipocytes, osteoblasts, and chondroblasts was identified using the tri-lineage differentiation of MSCs kit (CHEM-2000, Chembio Biotechnology Co., Ltd, Shanghai, China). The adipogenic differentiation reagent was oil red O (CHEM-200002-I); the osteogenic differentiation reagent was alizarin red (CHEM-200010-G) and the chondrogenic differentiation reagent was alcian blue (CHEM-200015-1).

### Isolation and identification of HucMSCs-EVs

FBS was ultra-centrifuged at 100,000 × g for 8 h to remove EVs in serum. The medium supernatant was removed when the HucMSCs confluence reached 80%. Following PBS washing twice, the cells were cultured with 10% FBS medium free of EVs at 37 °C with CO_2_ for 48 h. The collected supernatant was subjected to several centrifugations (300×*g* and 4 °C for 10 min; 2000×*g* and 4 °C for 15 min; 5000×*g* and 4 °C for 15 min; 12,000×*g* and 4 °C for 30 min) to remove the precipitate. Following PBS washing, the suspension was subjected to several centrifugations (12,000×*g* at 4 °C for 70 min; 100,000×*g* at 4 °C for 70 min; 100,000×*g* at 4 °C for 70 min) to collect the precipitate. HucMSCs were incubated in the 10% EVs-free FBS medium supplemented with GW4869 (Sigma-Aldrich, Merck KGaA, Darmstadt, Germany), and the conditioned medium was used as control (GW). EVs were treated with Rnase I (Thermo Fisher Scientific Inc., Waltham, MA, USA) and heat-inactivated, followed by the detection of miR-29b-3p expression in EVs.

The morphology of EVs was observed under transmission electron microscopy (TEM). The particle size distribution of EVs was measured using the NanoSight nanoparticle tracking analyzer (NTA; Malvern Instruments, Worcestershire, UK). The surface markers of EVs were identified using Western blotting. The total protein content of concentrated EVs suspension was determined using bicinchoninic acid (BCA) kit (23227, Thermo Fisher Scientific). The protein was separated on 10% sodium dodecyl sulfate-polyacrylamide gel electrophoresis and transferred onto membranes. Then, EVs specific marker proteins [tumor susceptibility gene 101 (TSG, 1:1000, ab83), CD63 (1:1000, ab134045) and CD9 (1:2000, ab92726)] and the negative control [GRP94 (1:1000, ab52031)] were detected.

### HucMSCs transfection

HucMSCs were transfected with miR-29b-3p inhibitor and NC (Genepharma, Shanghai, China) using Lipofectamine 2000 (Invitrogen Inc., Carlsbad, CA, USA) and cultured at 37 °C for 4 h. After the cells were cultured in a complete medium for another 48 h, EVs-inhibitor and EVs-NC were isolated.

### Establishment of a rat model of SCI

Adult specific pathogen-free grade Sprague Dawley (SD) rats (male, 150–200 g) were bought from Beijing Vital River Laboratory Animal Technology Co., Ltd (Beijing, China) and anesthetized with 3% sodium pentobarbital (P3761, Sigma-Aldrich). After paravertebral muscles were dissected, laminectomy was performed from T9 to T11. Then, SCI rats were inflicted with an aneurysm clip for 60 s at the T10. The incision was sutured in layers with silk suture. Sham-operated rats received laminectomy only (sham group). After the operation, all the rats were injected with penicillin and analgesics for 3 days and urinated artificially 3 times/day. At 24 h after the operation, the rats were assigned to 7 groups.

The rats were injected with 100 mg EVs (EVs group, EVs-NC group, and EVs-inhibitor group) or equivalent GW4869 conditioned medium of HucMSCs (GW group) via tail vein [57]. EVs suspension or PBS (SCI group) was injected into rats in the same coordinates with a Hamilton syringe once a week. In addition, SCI rats co-treated with EVs and PTEN adenovirus were assigned into EVs + AdNC group [rats were injected with 100 mg EVs and 60 μL (2 × 10^9^ pfu/mL) AdNC] and EVs + AdPTEN group [rats were injected with 100 mg EVs and 60 μL (2 × 10^9^ pfu/mL) AdPTEN]. Adenovirus vector of PTEN and its NC were purchased from Addgene (Cambridge, MA, USA). A total of 240 SCI model rats were used in this study, 30 rats in each group (Basso, Beattie & Bresnahan locomotor rating scale (BBB)) score and footprint analysis were performed in all rats; six rats were used for hematoxylin and eosin (HE) staining; six rats were used for Nissl staining; six rats were used for apoptosis detection; six rats were used for immunohistochemistry; six rats were used for reverse transcription-quantitative polymerase chain reaction (RT-qPCR) and Western blot analysis after tissue homogenization. On the 28th day after the operation, the spinal cord tissues were collected for related detection.

### In vivo observation of EVs

EVs were pre-labeled with PHK26 (Sigma-Aldrich) and incubated in the dark at 37 °C for 15 min. Then, EVs were centrifuged at 16,000×*g* for 60 min to remove the supernatant. Following PBS rinsing three times, the labeled EVs were injected into rats. EVs in vivo were observed under an Olympus BX41 microscope equipped with a charge-coupled device camera (Magnafire, Olympus, Tokyo, Japan) [58].

### BBB score

Behavioral tests of rats were performed on the 7th, 14th, 21st, and 28th days after operation [59]. The motor function of the hindlimb of rats was evaluated by the BBB scoring system. The rats were placed in an open field (125 cm × 125 cm), and their motor function was observed and evaluated when the rats were adapted. The observation (about 5 min) was performed with a double-blind method and scored by two non-experimenters who were familiar with the BBB quantification score at the same time. The average value of three recorded values was regarded as BBB score. Generally speaking, 0 score for complete paralysis and 21 scores for normal movement. The scores between 1 and 20 indicated the corresponding motor function of the hind limbs of the rats.

### HE staining

On the 28th day after the operation, the spinal cord tissues (0.5 cm above and below the center of the injury) were routinely dehydrated, paraffin-embedded, and sectioned. After that, the sections were dewaxed with xylene twice (5 min/time), dehydrated with gradient ethanol solution (100% for 5 min, 95% for 3 min, and 90% for 3 min), washed with water for 3 times, and stained with hematoxylin (Beyotime Biotechnology Co., Ltd, Shanghai, China) for 5 min. Subsequently, the sections were washed 3 times, differentiated with hydrochloric acid and alcohol for 20 s, and stained with 1% eosin for 5 min. The histological changes of spinal cord tissues were observed under a light microscope.

### Nissl staining

The sections were stained with 1% cresyl violet acetate (Solarbio Science & Technology Co., Ltd, Beijing, China) for 10 min. Then the sections were conventionally dehydrated with gradient ethanol, cleared with xylene, and sealed with neutral resin. The sections of 3–5 rats in each group were selected for statistical analysis. The number of viable neurons was observed and analyzed under a light microscope.

### Immunohistochemistry

Immunohistochemistry was conducted using the streptavidin peroxidase immunohistochemical kit (HSP0001, Shanghai MaiBio Biotechnology Co., Ltd., Shanghai, China). Each section was incubated in 3% H_2_O_2_ for 10 min to eliminate endogenous peroxidase activity and blocked with normal goat serum working solution (Beijing Kangwei Century Biotechnology Co., Ltd., Beijing, China). Afterward, the sections were cultured with NeuN (1:100, ab177487), NF-200 (1:100, ab215903), and glial fibrillary acidic protein (GFAP) (1:100, ab68428) at 4 °C overnight. The sections were washed with PBS 3 times and incubated with biotin goat anti-mouse (sheep anti-rabbit) IgG (Wuhan Boster Biological Technology Co., Ltd, Wuhan, Hubei, China) at 37 °C for 20 min. Following PBS washing 3 times (3 min/time), the sections were incubated with peroxidase-labeled streptavidin-biotin at 37 °C for 30 min. Freshly prepared 2,4-diaminobutyric acid (DA1010-3ml, Solarbio) was added to each section for development for 5–10 min, and the staining process was monitored under a microscope [60]. The expression was calculated by the percentage of positive cells in 1000 cells. Each density was quantified using Image-Pro Plus software.

### TUNEL staining

A one-step TUNEL assay kit (Roche, Mannheim, Germany) was used to detect the apoptosis of spinal cord tissue on the 28th day after the operation. Briefly, the apoptotic rate was expressed by the number of positive staining cardiomyocytes/the total cardiomyocytes × 100%. Nikon ECLIPSE Ti microscope (Nikon, Tokyo, Japan) was used for observation.

### RT-qPCR

Total RNA was extracted by TRIzol reagent (Invitrogen) and miR was extracted using mirVana PARIS kit (Ambion, Austin, Texas, USA). Prime Script RT kit (Takara, Dalian, China) was used for reverse transcription. Fluorescence quantitative PCR was performed using SYBR^®^ Premix Ex Taq^TM^ II kit (Takara) and ABI StepOne real-time PCR System (Applied Biosystems, Inc., Carlsbad, CA, USA), with glyceraldehyde-3-phosphate dehydrogenase (GAPDH) or U6 as the internal reference. The primers are shown in Table 1.

**Table 1:** Primer sequences.

Genes	Primer sequences
miR-29b-3p	F: 5′–TAGCACCATTTGAAATCAGTGTT–3′
	R: 5′–AACACTGATTTCAAATGGTGCTA–3′
PTEN	F: 5′–AATTTTTAAAGGCACAAGAGG–3′
	R: 5′–CCAGGAAGAGGAAAGGA–3′
U6	F: 5′–CCAGGAAGAGGAAAGGA–3′
	R: 5′–TATGGAACGCTTCACGAATTTG–3′
GAPDH	F: 5′–GAGAGACCCCACTTGCTGCCA–3′
	R: 5′–GGAAGAAGTTCCCATCGTCA–3′

### Western blot analysis

The total protein of tissues was extracted using radio-immunoprecipitation assay lysate (strong) (R0010, Solarbio). The protein concentration was determined using the BCA kit (20201ES76, Yeasen Company, Shanghai, China). The protein was separated on polyacrylamide gel electrophoresis and transferred onto the polyvinylidene difluoride membrane. After that, the membrane was blocked with 5% bovine serum albumin for 1 h and incubated with the following diluted primary antibodies at 4 °C overnight: PTEN (1:5000, ab154812, Abcam), Cleaved caspase-3 (1:500, ab13847, Abcam), Bax (1:500, ab53154, Abcam), Bcl-2 (1:1000, ab196495, Abcam), AKT (1:500, ab38449, Abcam), pAKT (1:1000, #9271, Cell Signaling Technology, Beverly, MA, USA), mTOR (1:10,000, ab134903, Abcam), pmTOR (1:1000, #2971, Cell Signaling Technology), and β-actin (1:5000, ab8226, Abcam). Following tris-buffered saline-tween buffer washing (5 min × 3 times), the membrane was added with horseradish peroxidase-labeled goat anti-rabbit IgG (ab205718, 1:20,000, Abcam). The membrane was added with enhanced chemiluminescence for development. ImageJ 1.48 u software (National Institutes of Health) was used for protein quantitative analysis, and the grayscale ratio of each protein to internal reference β-actin was used for protein quantitative analysis. The experiment was repeated three times.

### Statistical analysis

Data analysis was introduced using SPSS 21.0 (IBM Corp., Armonk, NY, USA). Kolmogorov–Smirnov method checked the data were in the normal distribution. Data are expressed as mean ± standard deviation. The independent-sample *t* test was adopted for analysis of comparisons between two groups. One-way analysis of variance (ANOVA) or two-way ANOVA was employed for the comparisons among multi-groups, followed by Tukey’s multiple comparison test. G*Power 3 [61] was used to determine the sample size needed to detect an effect with desired power. The *p* < 0.01 meant a statistically significant difference.

## Data Availability

The data that support the findings of this study are available from the corresponding author upon reasonable request.
